# Low, rather than High, Body Mass Index Is a Risk Factor for Acute Kidney Injury in Multiethnic Asian Patients: A Retrospective Observational Study

**DOI:** 10.1155/2018/3284612

**Published:** 2018-01-09

**Authors:** Allen Yan Lun Liu, Jiexun Wang, Milind Nikam, Boon Cheok Lai, Lee Ying Yeoh

**Affiliations:** ^1^Division of Renal Medicine, Department of General Medicine, Khoo Teck Puat Hospital, Singapore; ^2^Clinical Research Unit, Khoo Teck Puat Hospital, Singapore; ^3^Fresenius Medical Care Pte. Ltd., Singapore

## Abstract

**Background:**

Acute kidney injury (AKI) is common in hospitalised patients. The relationship between body mass index (BMI) and the risk of having AKI for patients in the acute hospital setting is not known, particularly in the Asian population.

**Methods:**

This was a retrospective, single-centre, observational study conducted in Singapore, a multiethnic population. All patients aged ≥21 years and hospitalised from January to December 2013 were recruited.

**Results:**

A total of 12,555 patients were eligible for the analysis. A BMI of <18.5 kg/m^2^ was independently associated with the development of AKI in hospitalised patients (odds ratio (OR): 1.23 [95% confidence interval [CI]: 1.04–1.44, *P* = 0.01]) but not for overweight and obesity. Subgroup analysis further revealed that underweight patients aged ≥75 and repeated hospitalisation posed a higher risk of AKI (OR: 1.25 [CI: 1.01–1.56], *P* = 0.04; OR: 1.23 [CI: 1.04–1.44], *P* = 0.01, resp.). Analyses by interactions between different age groups and BMI using continuous or categorised variables did not affect the overall probability of developing AKI.

**Conclusions:**

Underweight Asian patients are susceptible to AKI in acute hospital settings. Identification of this novel risk factor for AKI allows us to optimise patient care by prevention, early detection, and timely intervention.

## 1. Introduction

Acute kidney injury (AKI) is common in hospitalised patients. Depending on the definitions used and populations under study, 10–30% of hospitalised patients are admitted for or with AKI [1]. The use of AKI definitions from the Kidney Disease: Improving Global Outcomes (KDIGO) clinical practice guideline provided consensus for researchers to identify and stratify populations at risk [2]. 

The proportion of hospitalised patients with high body mass index (BMI, calculated as kg/m^2^) is increasing (up to 30% in developed countries) [3]. High BMI or obesity in the general population is notorious for its negative impact on morbidity and mortality. Obesity is associated with multiple comorbidities; hence, obese patients are postulated to have a higher risk of developing AKI. In fact, previous studies focusing on postsurgery and critically ill patients demonstrated that obese patients were more likely to develop AKI [4–8]. On the contrary, recent reports suggested an inverse or “U” shaped relationship between BMI and mortality in AKI patients [6, 7]. These studies highlighted that malnutrition especially in Asians, which is commonly associated with underweight, was independently associated with increased risks of AKI, morbidity, and mortality [9–11].

No observational studies have yet been specifically designed to explore the association between BMI and AKI in the acute care setting. The current literature has limited information on the Asian-specific BMI classification and its association with AKI risk [12, 13]. Therefore, we conducted this study to address the association between BMI and AKI risk in patients in acute hospital settings from multiethnic Asian backgrounds and to elucidate its biological plausibility for potential early interventions and treatment.

## 2. Materials and Methods

This was a single-centre, retrospective cohort study. All patients aged ≥21 years who were hospitalised at Khoo Teck Puat Hospital (a 550-bed regional general hospital) in Singapore, a multiethnic Asian country, from January to December 2013, were recruited for analysis. The study was approved by the National Health Group Domain Specific Review Board (DSRB) of Singapore with adherence to the Declaration of Helsinki. These patients were identified from the hospital's electronic medical records. Data collection included baseline demographics, BMI (calculated from height and weight recorded on hospital admission before any AKI occurrence), primary diagnoses for admission (by the International Classification of Diseases, Ninth Revision [ICD-9] codes), background diagnoses, surgical procedures, laboratory data, hospitalisation length of stay, and survival status within 90 days upon discharge. We excluded individuals with pregnancy and end stage renal failure requiring either maintenance dialysis or renal transplantation. We also excluded patients without measurement of BMI or those with seemingly erroneous measurements (height <70 cm or >250 cm; weight >200 kg). We used an enzymatic colorimetric assay from Custom Biotech, Roche®, for measurement of serum creatinine. AKI was defined by the serum creatinine based KDIGO classification system [2], whereby AKI is defined as a ≥1.5-fold increase in serum creatinine within the previous 7 days, or ≥26.5 *μ*mol/l (≥0.3 mg/dl) increase from baseline within 48 hours. Baseline creatinine was defined as the median of all creatinine values obtained within 12 months preceding the occurrence of AKI for the index admission. If no previous serum creatinine was available, or only one serum creatinine result was known during hospital stay, the baseline creatinine was estimated by backward calculation from the simplified Modification of Diet in Renal Disease (MDRD) formula (assuming a glomerular filtration rate [GFR] of 75 ml/min per 1.73 m^2^) [14]. The median time from baseline creatinine measurement to hospitalisation was 71 days. A median of 4 creatinine measurements was used for the determination of the baseline value in our cohort (Tables 1 and 3). We determined the severity of AKI according to the KDIGO criteria. The AKI stages were calculated using serum creatinine with reference to baseline creatinine levels, or using estimated GFR. In order to validate our findings from estimated GFR by MDRD formula, we performed sensitivity analysis for a different AKI definition based on baseline creatinine. Cohen's kappa measured the degree of agreement by these two definitions of AKI, and the result was interpreted by Landis et al.'s guidelines [15, 16]. When we tested the agreement of AKI status between the use of creatinine based on MDRD estimations (if baseline creatinine was not known) and that based on true baseline creatinine values, we had 275 AKI patients (17.1% of the AKI group) accounting for the discordance, giving rise to a Cohen's kappa of 0.674. The agreement was good according to Landis et al.'s guidelines [15, 16]. We did not use urine output as part of the definition of AKI as the information was not available from the electronic records. The BMI was categorised based on the World Health Organisation (WHO) recommendations for Asians [12] (underweight [BMI < 18.5 kg/m^2^], normal [18.5–23 kg/m^2^], overweight [23–27.5 kg/m^2^], and obese [>27 kg/m^2^]). The incidence of AKI was then calculated for each group of the BMI. We used multivariate linear and nonlinear regression analyses for risk factor identification. We also tested for interactions among different variables against BMI to determine any existence of effect modifiers influencing the relationship between AKI and BMI.

## 3. Statistical Analysis

Continuous variables were summarised as means and standard deviations if normally distributed, and medians and quartiles if distributions were skewed. Categorical variables were presented as frequencies and percentages. We compared clinical and baseline characteristics of patients with and without AKI by using Chi-square test or Fisher's exact test for categorical variables. Student's* t*-test and Mann–Whitney* U* test were used for continuous variables with and without normal distribution, respectively (some continuous variables, e.g., age, were expressed as categorical variables with reference to previous published study designs and recommendations) [17, 18]. Statistically significant variables were considered as potential risk factors associated with the patients' AKI status. The bootstrap method is one of the popular methods for selecting the best subset of variables and developing parsimonious prediction models [19]. We referred to a previous study of similar recruitment scale and randomly generated 200 bootstrap datasets with the same size (*N* = 12,555) from the original data [20]. For each dataset, we performed multivariable logistic regression with a stepwise procedure to select independent risk factors associated with AKI status as the outcome. Variables that were selected in at least 90% of the bootstrap procedures were the best subset of potential risk factors and were included in the final multivariable logistic regression. We used Hosmer-Lemeshow's test for evaluating model goodness of fit. The model fitted the data well if *P* value ≥ 0.05. We further constructed sets of logistic regression models and performed comparison by the Chi-square test with the primary model if interactions were suspected among different variables which had confounding effects not detected by the primary model.

To delineate the relationship between AKI and BMI, if an assumption of nonlinear relation between continuous BMI and log odds of having AKI was made instead of categorisation, we further developed different models by means of restricted cubic splines (RCS) as a continuous natural spline regression. Model fitness of RCS regression was determined by choice of the number of knots (*k*) in comparison to cutoffs by BMI categories [21, 22]. We used *k* = 5 which was considered as adequate with a sample size of more than a hundred [23]. The advantage of RCS regression was that it modeled a wide range of possible nonlinear relations between BMI and AKI while keeping a good balance between model goodness of fit and simplicity (i.e., model parsimony) [23].

A *P* value of < 0.05 was considered statistically significant. All statistical analyses were performed with R Version 3.3.1 and SPSS® Version 22 (IBM® Corporation).

## 4. Results

**Figure 1:**
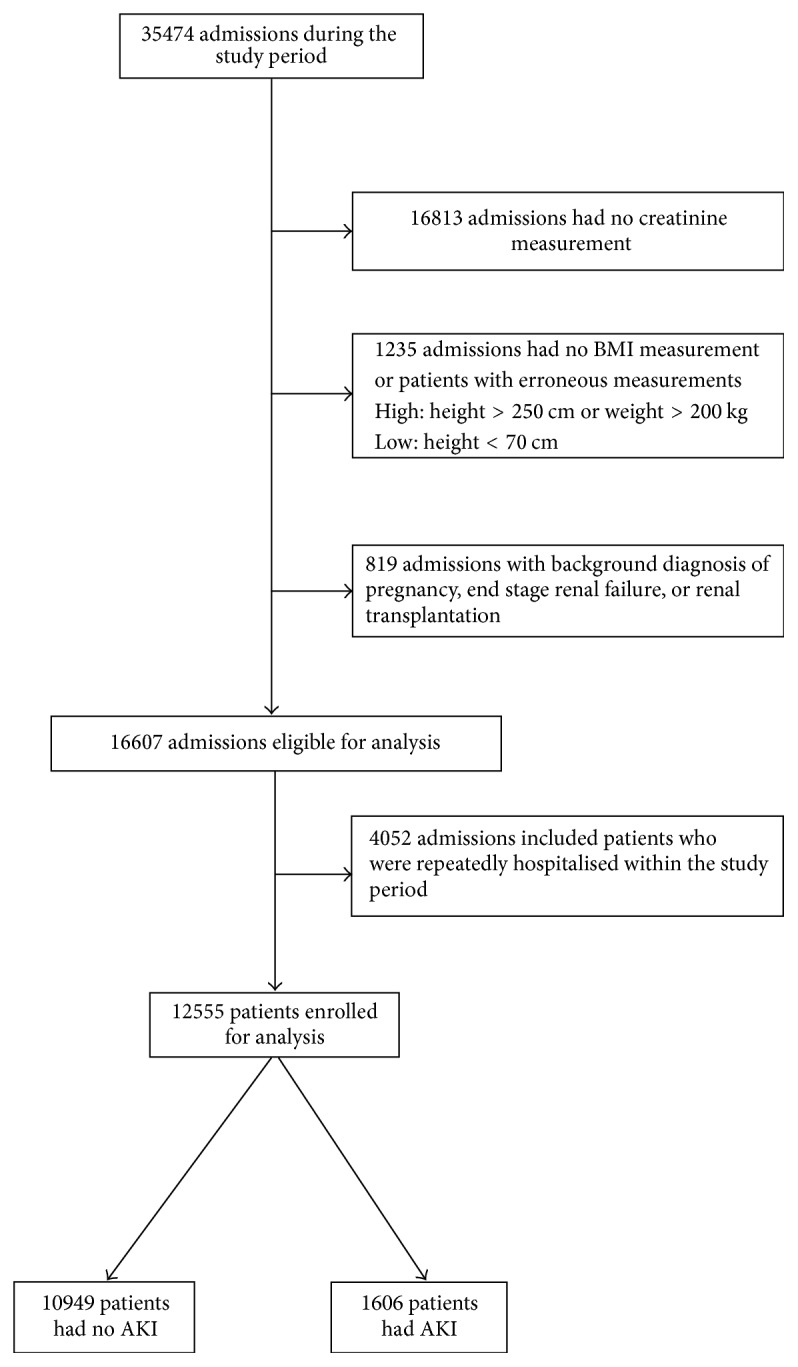
Flowchart of patient selection.

A total of 35,474 admissions were recorded during the study period. The patients with repeated admissions during the study period that had their admissions with the highest AKI staging were selected. As a result, 12,555 patients were eligible for analysis.  Figure 1 shows the flowchart of patient selection for data analysis.* Baseline characteristics of excluded patients are presented in the Supplementary Material* (available here). In our cohort, 61.9% were of a Chinese ethnicity and 53.8% were men. The median age was 64 years (interquartile range [IQR]: 51–77). Seventy-five percent of the patients were hospitalised under the care of general medical units or related specialties, namely, cardiology, nephrology, geriatrics, endocrinology, respiratory medicine, and gastroenterology. ICU admissions comprised 13% of the cohort, while 7.9% of the cohort succumbed to death within 90 days of hospital admission. The most common background diagnosis was hypertension (39%), followed by dyslipidemia (31%), diabetes mellitus (26.2%), and cardiovascular disease (20%).

### 4.1. The Incidence of AKI for Each Category of BMI

1,606 patients developed AKI in our cohort (incidence of 12.8%). The severity of AKI was defined as per the KDIGO guidelines (AKI stage 1 (AKI1), defined as a 1.5–1.9-fold increase in serum creatinine within the previous 7 days, or ≥26.5 *μ*mol/l (≥0.3 mg/dl) increase from baseline within 48 hours, comprised 5.5% of the cohort (or 42.7% of the AKI group); AKI stages 2 (AKI2) and 3 (AKI3), defined as 2.0–2.9-fold and ≥3-fold increase in serum creatinine from baseline within 7 days, or the need for dialysis, or when serum creatinine ≥353.6 *μ*mol/l (≥4.0 mg/dl), resp., had an incidence of 3.8% and 3.5% for the whole cohort or 29.6% and 27.7% for the AKI group, resp.). The patients who had recurrent admissions over the 12-month period comprised 33% of the cohort.

### 4.2. Characteristics among Different BMI Categories

Baseline characteristics, length of stay, need for intensive care, 90-day mortality, time from baseline creatinine measurement to hospitalisation, and number of creatinine measurements within 12 months before hospitalisation are shown in Table 1 according to BMI categorisation. More obese patients compared to normal BMI patients required ICU admissions (*P* < 0.0001). On the contrary, patients with low BMI had higher mortality within 90 days of admission (*P* < 0.0001).

### 4.3. Factors Associated with AKI

**Figure 2:**
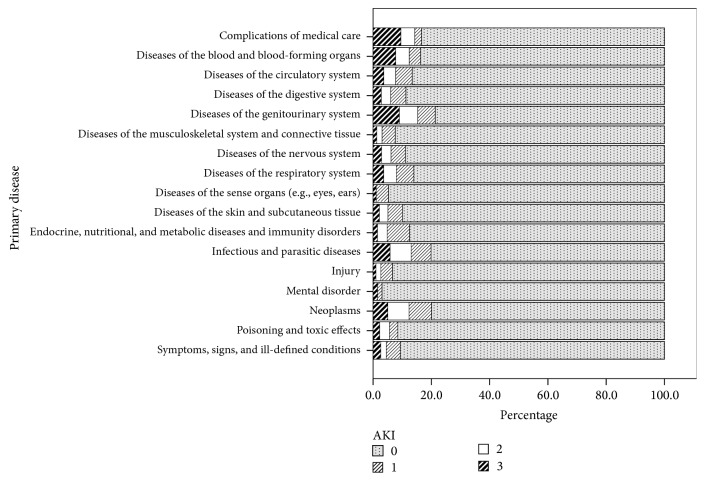
Acute kidney injury categories according to KDIGO classification by percentages according to the primary diagnosis by ICD-9 codes. AKI: acute kidney injury, defined as a ≥1.5-fold increase in serum creatinine within the previous 7 days, or ≥26.5 *μ*mol/l (≥0.3 mg/dl) increase from baseline within 48 hours; AKI1: 1.5–1.9-fold increase in serum creatinine within the previous 7 days, or ≥26.5 *μ*mol/l (≥0.3 mg/dl) increase from baseline within 48 hours; AKI2: 2.0–2.9-fold increase in serum creatinine within the previous 7 days; AKI3: 3-fold or higher increase in serum creatinine from baseline within 7 days, or the need for dialysis, or when serum creatinine ≥ 353.6 *μ*mol/l (≥4.0 mg/dl).

The primary diagnoses in our cohort with or without AKI are listed in Table 2. Two important contributing causes for AKI were infection and neoplasm.  Figure 2 shows further categorisation of AKI according to ICD-9 codes, among which AKI severity varied with different primary diagnoses. Baseline characteristics, length of stay, need for intensive care, 90-day mortality, time from baseline creatinine measurement to hospitalisation, and number of creatinine measurements within 12 months before hospitalisation are shown in Table 3 according to AKI status. With reference to normal BMI (18.5–23 kg/m^2^), the unadjusted odds ratio (OR) for the risk of AKI in patients with BMI < 18.5 kg/m^2^ was 1.27 (95% confidence interval [CI]: 1.08–1.48; *P* = 0.003).

### 4.4. Association between AKI and BMI

**Figure 3:**
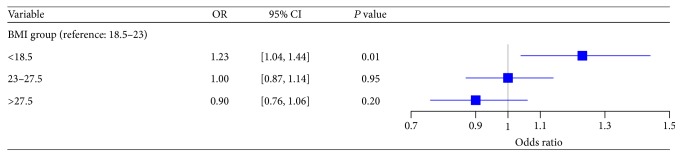
Multivariate logistic regression models for studying the relationship between BMI and acute kidney injury in hospitalised patients (total number of patients = 12,555; patients with acute kidney injury = 1606; patients with no acute kidney injury = 10,949).* Note*. BMI: body mass index; CI: confidence interval; OR: odds ratio. Adjusted variables in logistic regression models include age, gender, ethnicity, background diagnoses (cardiovascular disease, chronic kidney disease, diabetes mellitus, hepatobiliary disease, psychiatric illness, and gastrointestinal disease), primary diagnoses (infectious and parasitic diseases, neoplasm, diseases of the genitourinary system, diseases of the musculoskeletal system and connective tissue, and symptoms, signs, and ill-defined conditions), and procedures (otolaryngology related surgery, endoscopy, open laparotomy, and cardiology related percutaneous procedures).

Table 4 shows the variables selected from the bootstrap method for multivariate logistic regression modeling (except for age, gender, ethnicity, and BMI). We identified several traditional background diagnoses that were independently associated with the risk of AKI (diabetes mellitus, cardiovascular disease, and CKD). History of hepatobiliary disease and psychiatric illness was also associated with an increased risk of AKI. The risk of AKI from infectious diseases was significantly higher for patients aged 55 years or older when compared to younger populations (OR for age 55–75: 2.35 [95% CI: 1.72–3.19]; OR for age > 75: 2.51 [95% CI: 1.90–3.30]). Patients that underwent procedures like endoscopy, open laparotomy, and surgeries related to otolaryngology showed a higher risk of AKI, while percutaneous cardiovascular procedures revealed the opposite. After adjusting the risk factors shown in Table 4 (age, gender, ethnicity, background diagnoses, primary diagnoses, and procedures), BMI of <18.5 kg/m^2^ remained independently associated with the development of AKI in hospitalised patients (OR: 1.23 [95% CI: 1.04–1.44], *P* = 0.014), while overweight (BMI: 23–27.5 kg/m^2^) and obese (BMI > 27.5 kg/m^2^) patients did not show any significant associations (OR 1.00 for overweight [95% CI: 0.87–1.14]; OR 0.90 for obesity [95% CI: 0.76–1.06]; see Figure 3).

**Figure 4:**
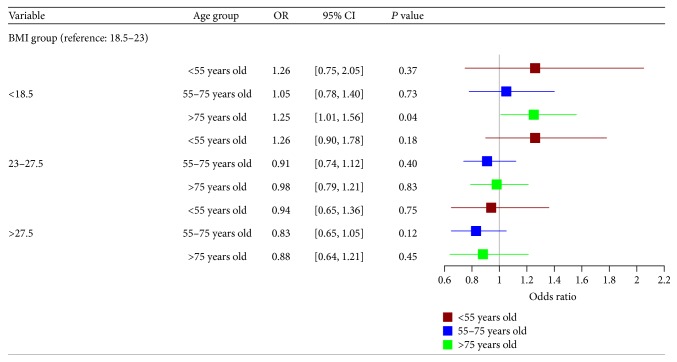
Multivariate logistic regression models for studying the relationship between body mass index (BMI) and acute kidney injury (AKI) for different age groups of hospitalised patients (total number of patients under 55 years of age = 3960, where patients with AKI = 259; total number of patients aged 55–75 years = 4796, where patients with AKI = 681; total number of patients above 75 years old = 3799, where patients with AKI = 666).* Note*. BMI: body mass index; CI: confidence interval; OR: odds ratio. Adjusted variables in logistic regression models include age, gender, ethnicity, background diagnoses (cardiovascular disease, chronic kidney disease, diabetes mellitus, hepatobiliary disease, psychiatric illness, and gastrointestinal disease), primary diagnoses (infectious and parasitic diseases, neoplasm, diseases of the genitourinary system, diseases of the musculoskeletal system and connective tissue, and symptoms, signs, and ill-defined conditions), and procedures (otolaryngology related surgery, endoscopy, open laparotomy, and cardiology related percutaneous procedures).

**Figure 5:**
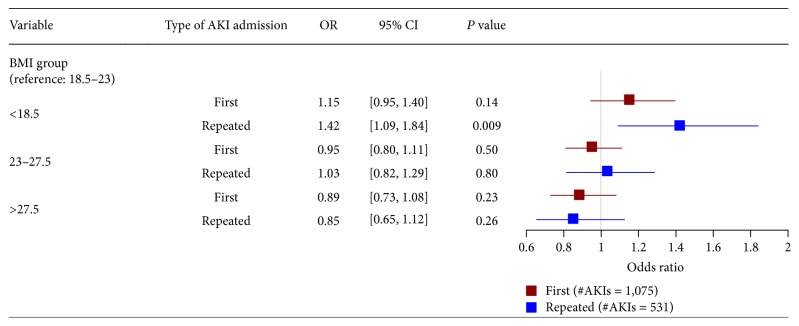
Multivariate logistic regression models for studying the relationship between BMI and acute kidney injury at their first (number of patients with acute kidney injury [#AKIs] = 1075) and repeated (number of patients with no acute kidney injury [#AKIs] = 531) admissions during the 12-month cohort period.* Note*. BMI: body mass index; CI: confidence interval; OR: odds ratio. Adjusted variables in logistic regression models include age, gender, ethnicity, background diagnoses (cardiovascular disease, chronic kidney disease, diabetes mellitus, hepatobiliary disease, psychiatric illness, and gastrointestinal disease), primary diagnoses (infectious and parasitic diseases, neoplasm, diseases of the genitourinary system, diseases of the musculoskeletal system and connective tissue, and symptoms, signs, and ill-defined conditions), and procedures (otolaryngology related surgery, endoscopy, open laparotomy, and cardiology related percutaneous procedures).

Subgroup analyses revealed a significant increase in the risk of AKI for patients aged >75 and those with BMI < 18.5 kg/m^2^ (OR: 1.25 [95% CI: 1.01–1.56]), while the rest of the study population did not reveal similar findings (Figure 4). We also found that underweight patients who had recurrent admissions were predisposed to a higher risk of AKI (OR: 1.42 [95% CI: 1.09–1.84]; see Figure 5).

In order to distinguish the AKI risk among different age groups, we further tested the logistic model by adding interactions for age groups and BMI categories (i.e., addition of all possible combinations for individual age group* times* BMI categories, e.g., BMI < 18.5 kg/m^2^  × age < 55 years). The possible interactions derived from all the crossovers were not statistically significant (Chi-square test = 5.58; *P* = 0.47), which implied that age was not an effect modifier for BMI and AKI.

**Figure 6:**
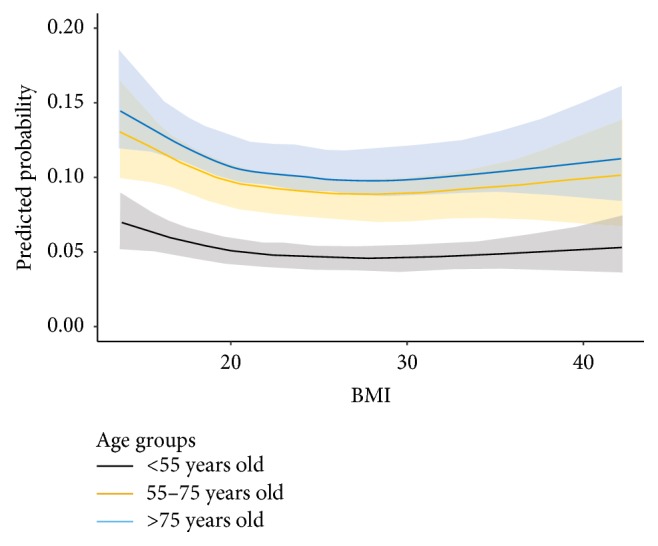
The nonlinear relation between continuous BMI and the probability of having AKI between different age groups by restricted cubic spline regression (total number of patients = 12,555; patients with acute kidney injury = 1606; patients with no acute kidney injury = 10,949).* Note*. BMI: body mass index; 95% confidence intervals (CIs) are denoted by a grey shadow. Adjusted variables in logistic regression models include age, gender, ethnicity, background diagnoses (cardiovascular disease, chronic kidney disease, diabetes mellitus, hepatobiliary disease, psychiatric illness, and gastrointestinal disease), primary diagnoses (infectious and parasitic diseases, neoplasm, diseases of the genitourinary system, diseases of the musculoskeletal system and connective tissue, and symptoms, signs, and ill-defined conditions), and procedures (otolaryngology related surgery, endoscopy, open laparotomy, and cardiology related percutaneous procedures).

To evaluate the relationship between AKI and BMI in a continuous manner, we built up RCS regression models based on 5 knots (i.e., 5%, 25%, 50%, 75%, and 95% percentiles of BMI) and 3 alternative prespecified knots (BMI = 18.5 kg/m^2^, 23 kg/m^2^, and 27.5 kg/m^2^, same as cutoffs for BMI categorisation). RCS using 5 knots had a very similar predicted probability of having AKI when compared with the primary logistic regression model as shown in Table 4 (correlation coefficient = 0.99, *P* < 0.001). RCS with the 3 prespecified knots' model also yielded a similar result (correlation coefficient = 1.0, *P* < 0.001). We repeated RCS using 5 knots with the addition of individual age group* times* BMI categories with crossover, again revealing negative interactions between age and BMI (Chi-square test = 13.18; *P* = 0.11).  Figure 6 demonstrates the continuous relationship between BMI and the probability of having AKI stratified by age groups (adjusted with gender, ethnicity, background diagnoses, primary diagnoses, and procedures). The CIs of the odds ratio for the group of patients below 55 years of age do not overlap with those CIs for the group of patients >75 years old. This means that, compared to patients <55 years old, the effect of BMI on the occurrence of AKI is statistically significantly different from those patients >75 years old. We had a similar observation for patients aged 55–75 years whose BMI <40. However, we were able to determine whether there is a significant difference in the effect of BMI on AKI between patients 55–75 years of age and patients >75 years of age due to their overlapped CIs. Overall, the adjusted RCS plot showed that, given the same BMI, different age groups had very similar differences in terms of the probability of developing AKI.

## 5. Discussion

### 5.1. Summary of Findings

This retrospective cohort study included 35,474 admissions to a general hospital serving a multiethnic population from January to December 2013. The incidence of AKI by KDIGO classification was 12.8%. We found that the overall risk of AKI was significantly higher in patients who were underweight (BMI < 18.5 kg/m^2^) compared to normal BMI according to the WHO guidelines for Asians (18.5–23 kg/m^2^). These findings were more prominent in the elderly population aged >75 who had repeated hospitalisation. However, multivariate analyses did not show any significant interaction between age group and BMI for the risk of developing AKI. The logistic regression and RCS analyses in addition to interactions between different age groups and BMI using categorised or continuous variables, respectively, did not affect the probability of developing AKI. The association between low BMI and AKI risk remained significant after adjusting for gender, comorbidities, primary diagnoses for respective hospital admissions, and procedures or surgical interventions, thus further highlighting the strength of this association.

### 5.2. Insights from Literature Review

Previous studies on the impact of BMI on AKI mainly focused on hospitalised surgical patients who underwent either cardiac [24] or noncardiac [4, 5, 25–27] surgery, or both medical and surgical patients with critical illnesses that required intensive care [6, 7, 28]. These studies consistently concluded the adverse effect of high BMI on the risk of AKI. The pathophysiologic pathway leading to AKI in obese surgical and critically ill patients was discussed in recent review articles [29–31]. The authors basically aligned with the observation on the adverse impact of high BMI on AKI, despite the physiological stress incurred during perioperative and postoperative periods together with hemodynamic instability when critically ill. In this study, we observed the reciprocal effect of BMI and AKI, where being underweight was associated with the risk of AKI.

This phenomenon is an interesting observation and makes one wonder whether multiethnic Asian hospitalised patients behave in a rather contradictory manner. In our study, we demonstrated several surgical interventions (endoscopy, cardiology related procedures, and open laparotomy) per se independently associated with the risk of AKI using the same logistic regression model involving BMI (Table 4). This observation parallels some studies whereas the effect of high BMI on AKI attenuated when concomitant risk factors were removed (e.g., CKD) [32] or accentuated when metabolic risk factors were inserted [25]. Thus, it remains unclear whether obesity is genuinely an independent risk factor for the development of AKI. Our study provides new insight into the relationship between BMI and AKI, whereby being underweight, rather than overweight, serves as an important yet undisclosed risk factor to kidney insult in hospitalised patients.

Information on the relationship between AKI and being underweight and likely malnourished was scarce [6, 33–35]. However, a paradoxical decrease in mortality and morbidity with an increase in BMI, referred to as the “obesity paradox,” has been readily observed in patients with CKD [36–41], patients on long-term dialysis [42], and geriatric population [43]. As shown in Figure 6, among obese patients, there was a statistically nonsignificant increased probability of AKI. This is consistent with the result from a multicentre observational study in Austria with the lowest risk of developing AKI in patients with normal body weight (a “U” shaped curve) [6]. Similar findings on AKI and mortality were reported in Asian populations [10, 35]. The apparent knowledge gap in high BMI exerts protective or at best nondetrimental effects on AKI in hospitalised patients that could be explained by several mechanisms. The protective effect of obesity from inflammation (one of the most important pathophysiologies in AKI) is partially contributed generally by a higher level of lipoproteins in obese patients with endothelial protection from endotoxins in the renal vasculature [44]. On the other hand, underweight patients with chronic illnesses mimic the “protein energy wasting” status, whereby high background inflammatory burden and/or inflammatory response is/are triggered in reaction to acute illness during hospitalisation [45, 46]. It is well established that infection as one of the manifestations in acute illnesses is notorious for being the major risk factor for AKI in hospitalised patients [47]. Of note, during acute illnesses with high catabolic rate and increase in energy consumption, obese patients could optimise the utilisation of stored lipids through ketogenesis as an alternate energy source, alleviating the risk of organ injury [42, 48]. In our study, this association was stronger for patients aged 55 years or above, which is consistent with the previous studies, whereas older populations with lower energy reserve are susceptible to developing AKI [9, 49]. Given the independent association between AKI and BMI (adjusted with infective causes by the ICD code and interactions among different age groups), interplay among AKI, inflammation, and malnutrition that are triggered by noninfective processes should require elucidation in the future studies.

Subgroup analysis found that elderly patients (aged > 75) who were underweight incurred a higher risk of AKI compared to those with normal BMI. This echoed those findings from previous studies on elderly patients that use postoperative AKI or mortality as outcome measures [11, 26]. In the context of BMI measurement, particularly in the elderly population, where central obesity is the predominant type for overweight in Asians compared to the western population [12, 50], it is impossible to distinguish subcutaneous from visceral adipose tissue component by this simple calculation. Visceral adiposity is notorious and responsible for the development of metabolic syndrome and CKD; both predispose to the risk of AKI [25, 51]. Anthropometric assessments such as waist circumference and waist-to-hip ratio seem to reflect better as surrogates for the extent of visceral adipose mass [52]. However, BMI remains the preferred choice of measurement for obesity as it is simple to apply and easily accessible for routine practice in all hospitalised patients and large-scale studies as demonstrated in our study.

Another important clinical finding from our cohort is that recurrent admissions for underweight patients have been associated with the risk of developing AKI. Recent studies revealed the strong associations between AKI and hospital readmission, both in surgical and in nonsurgical patients [53, 54]. There are many reasons for underweight patients requiring repeated hospitalisation, partly linked to more complex interplay between comorbidities and risks of infection [55]. On the other hand, patients who had multiple hospitalisations within a short period of time might incur a significant impact on the nutritional status as well. The sequel of independent association between underweight and AKI from our study after adjusting for the variables provided insights for future studies to delineate the exact relationship between these two clinical entities.

### 5.3. Clinical Relevance to the New Findings

To apply the findings from our current study in clinical practice, we aim at first to generalise the measurement of BMI to all hospitalised patients instead of critically ill or surgical patients alone, since we can easily implement recording of body weight and height in routine nursing practice upon every hospital admission, same as blood pressure and temperature measurements. This could then further translate into a preventive strategy for the development of AKI before its actual occurrence with a subsequent timely intervention. While, in view of being an observational study, associations cannot be deemed causal in nature, the study allows us to identify underweight, particularly elderly population with a higher chance of readmission as a potentially high-risk group. This in turn allows us to take extra precautions in the management, such as dose adjustments in therapeutic drug administration and preventive measures when using contrast, and to maintain the hemodynamic stability in cardiovascular disease and sepsis; such events are commonly encountered in hospitalised individuals.

### 5.4. Strengths and Limitations

To the best of our knowledge, this is the first study providing the association between underweight and higher risk of developing AKI among hospitalised patients from a multiethnic Asian background. Moreover, this study included a significantly diverse spectrum of hospital presentations—only excluding pediatric, obstetric, end stage renal failure, and posttransplant patients—thereby minimising the probability of selection bias. We used AKI classification from KDIGO as a reference since it was shown to be a more reliable system in AKI epidemiological studies than other classifications [56].

Nonetheless, the intrinsic fallacies of this study, including recruitment of a cohort from a single centre and predominance of ethnicity, render generalisability to other ethnicity groups questionable. Its retrospective and observational nature, hence the establishment of a causal relationship between AKI and BMI, is impossible. Like other AKI epidemiological studies, we did not have data on urine output and also baseline plasma creatinine levels if not known previously. Furthermore, we only recruited patients who had creatinine measurements performed. This can lead to selection bias wherein those patients with short hospitalisation, who experienced self-limiting AKI, may have been missed from the analysis. We measured baseline creatinine in both inpatient and outpatient settings (for baseline creatinine), or derived baseline creatinine from the MDRD formula, whereby it may misclassify AKI particularly in the critical care settings [57], and its estimation may also become less reliable in obesity [14]. We also had insufficient data on the nutritional and functional status for our cohort, namely, albumin and acute inflammatory markers. Moreover, we do not have data for those patients who underwent hospitalisation prior to the study period (patients with AKI at the “first” admission shown in Figure 5 might be repeatedly admitted if we stretched the timeline of observation prior to the current study period). Malnourished patients are more likely to have readmissions that lead to potential bias in our study. All these factors may interfere with the actual incidence of AKI and subsequent analysis. Nevertheless, given the large sample size in this study and the substantial agreement from sensitivity analysis between baseline creatinine and those estimated by MDRD formula, the above effects, if any, would have negligible influences on the study outcomes.

## 6. Conclusions

In this single-centre study involving 12,555 multiethnic Asians, we found a significant association between being underweight and the risk of having AKI in acute care settings. Measuring BMI for all the admissions is straightforward. Using this novel risk factor to detect the population at risk in addition to the conventional factors is important for prevention, early identification, and targeted care to minimise AKI and subsequent progression to chronic kidney disease. Further studies that involve different populations are needed to establish a causal relationship between being underweight and this important and common clinical entity.

## Figures and Tables

**Table 1:** Baseline characteristics in relation to body mass index categories according to the World Health Organisation (WHO) classification on Asians.

	BMI, kg/m^2^				
	Total	<18.5	18.5–23	23–27.5	>27.5
Number of patients	12555	1727	4339	3902	2587
*Demographics*					
Age, year	63 ± 18.5	70 ± 19.4	63 ± 19.5	62 ± 17.3	57 ± 15.9
Gender, men (%)	6755 (53.8)	856 (49.6)	2329 (53.7)	2264 (58.0)	1306 (50.5)
*Ethnicity (*%)					
Chinese	7776 (61.9)	1257 (72.8)	2942 (67.8)	2424 (62.1)	1153 (44.6)
Malay	2360 (18.8)	230 (13.3)	626 (14.4)	721 (18.5)	783 (30.3)
Indian	1485 (11.8)	146 (8.5)	458 (10.6)	460 (11.8)	421 (16.3)
Others	934 (7.4)	94 (5.4)	313 (7.2)	297 (7.6)	230 (8.9)
*Specialty (*%)					
Medicine	6353 (50.6)	871 (50.4)	2175 (50.2)	1977 (50.1)	1330 (51.4)
Surgery	1679 (13.4)	173 (10.0)	631 (14.5)	548 (14.0)	327 (12.6)
Geriatrics	1591 (12.7)	427 (24.7)	637 (14.7)	399 (10.2)	128 (4.9)
Cardiology	1479 (11.8)	111 (6.4)	417 (9.6)	503 (12.9)	448 (17.3)
Orthopedic surgery	1147 (9.1)	121 (7.0)	391 (9.0)	360 (9.3)	275 (10.6)
Urology	248 (2.0)	17 (1.0)	73 (1.7)	94 (2.4)	64 (2.4)
Otolaryngology	41 (0.3)	5 (0.3)	9 (0.2)	14 (0.4)	13 (0.5)
Ophthalmology	17 (0.1)	2 (0.1)	6 (0.1)	7 (0.2)	2 (0.1)
*Background diagnosis (*%)					
Hypertension	4895 (39.0)	653 (37.8)	1579 (36.4)	1519 (38.9)	1144 (44.2)
Dyslipidemia	3896 (31.0)	435 (25.2)	1229 (28.3)	1271 (32.6)	961 (37.1)
Diabetes mellitus	3289 (26.2)	341 (19.7)	1039 (23.9)	1072 (27.5)	837 (32.4)
Cardiovascular disease	2515 (20.0)	349 (20.2)	878 (20.2)	771 (19.8)	517 (20.0)
Orthopedic related disease	1402 (11.2)	262 (15.2)	470 (10.8)	397 (10.2)	273 (10.6)
Cerebrovascular disease	1314 (10.5)	256 (14.8)	485 (11.2)	392 (10.0)	181 (7.0)
Neurological disease	1219 (9.7)	283 (16.4)	496 (11.4)	307 (7.9)	133 (5.1)
Respiratory disease	1030 (8.2)	199 (11.5)	292 (6.7)	257 (6.6)	282 (10.9)
Hematological disease	955 (7.6)	230 (13.3)	380 (8.8)	226 (5.8)	119 (4.6)
Gastrointestinal disease	936 (7.5)	221 (12.2)	324 (7.5)	243 (6.2)	158 (6.1)
Chronic kidney disease	857 (6.8)	111 (6.4)	270 (6.2)	286 (7.3)	190 (7.3)
Psychiatric illness	760 (6.1)	172 (10.0)	290 (6.7)	205 (5.3)	93 (3.6)
Urological disease	595 (4.7)	120 (6.9)	238 (5.5)	157 (4.0)	80 (3.1)
Underlying malignancy	584 (4.7)	140 (8.1)	234 (5.4)	148 (3.8)	62 (2.4)
Hepatobiliary disease	463 (3.7)	64 (3.7)	155 (3.6)	147 (3.8)	97 (3.7)
*Procedures (*%)					
Endoscopy	2355 (18.8)	363 (21.0)	853 (19.7)	673 (17.2)	466 (18.0)
Orthopedic surgery	1222 (9.7)	170 (9.8)	413 (9.5)	368 (9.4)	271 (10.5)
Cardiology related procedures (non-open-heart surgery)	584 (4.7)	32 (1.9)	156 (3.6)	219 (5.6)	177 (6.8)
Excision of skin lesions	851 (6.8)	64 (3.7)	259 (6.0)	195 (7.6)	233 (9.0)
Open laparotomy	579 (4.6)	77 (4.5)	231 (5.3)	182 (4.7)	89 (3.4)
Urology related surgery	510 (4.1)	61 (3.5)	169 (3.9)	161 (4.1)	119 (4.6)
Laparoscopic surgery (intra-abdominal)	265 (2.1)	23 (1.3)	85 (2.0)	81 (2.1)	75 (2.9)
Neurosurgery	159 (1.3)	19 (1.1)	68 (1.6)	50 (1.3)	22 (0.9)
Otolaryngology related surgery	149 (1.2)	26 (1.5)	55 (1.3)	33 (0.8)	35 (1.4)
Vascular surgery, open	57 (0.5)	8 (0.5)	15 (0.3)	25 (0.6)	9 (0.3)
Laparoscopic assisted bariatric bypass surgery	12 (0.18)	0 (0)	0 (0)	5 (0.1)	7 (0.3)
*Hospitalisation characteristics*					
ICU admission (%)	1638 (13.0)	155 (9.0)	490 (11.3)	571 (14.7)	421 (16.3)
Mortality within 90 days of admission (%)	997 (7.9)	256 (14.8)	414 (9.5)	239 (6.1)	88 (3.4)
Length of stay (d, IQR)	5 (3–9)	7 (4–14)	5 (3–10)	4 (3–8)	4 (2–7)
*Creatinine measurements*					
Time from baseline measurement to hospitalisation (d, IQR)	71 (29–147)	62 (30–140)	69 (28–140.8)	69 (26–148.8)	79 (32–159)
Number of measurements within 365 days before hospitalisation (*n*, IQR)	4 (2–6)	4 (3–6)	4 (2–6)	4 (2–7)	2 (2–6)

AKI: acute kidney injury; ICU: intensive care unit; BMI: body mass index; IQR: interquartile range.

**Table 2:** Primary diagnosis of hospitalised patients with acute kidney injury according to ICD-9 codes and KDIGO classification.

Primary diagnosis	Total	No acute kidney injury (%)	Acute kidney injury (%)	*P* value
Total number	12555	10949	1606	
Diseases of the respiratory system	1625 (12.9)	1398 (12.8)	227 (14.1)	0.128
Symptoms, signs, and ill-defined conditions	1547 (12.3)	1404 (12.8)	144 (8.9)	<0.001
Diseases of the circulatory system	1505 (12.0)	1305 (11.9)	202 (12.5)	0.538

Diseases of the digestive system	1423 (11.3)	1263 (11.5)	160 (10.0)	0.063
Diseases of the musculoskeletal system and connective tissue	1104 (8.8)	1020 (9.3)	84 (5.2)	<0.001
Diseases of the nervous system	1029 (8.2)	915 (8.4)	114 (7.1)	0.086
Diseases of the genitourinary system	966 (7.7)	760 (6.9)	206 (12.8)	<0.001

Infectious and parasitic diseases	925 (7.4)	742 (6.8)	183 (11.4)	<0.001
Diseases of the skin and subcutaneous tissue	645 (5.1)	581 (5.3)	65 (4.0)	0.025
Endocrine, nutritional, and metabolic diseases and immunity disorders	536 (4.3)	469 (4.3)	67 (4.2)	0.836
Neoplasm	438 (3.5)	350 (3.2)	88 (5.5)	<0.001
Injury	303 (2.4)	283 (2.6)	20 (1.2)	0.001
Poisoning and toxic effects	177 (1.4)	162 (1.5)	15 (0.9)	0.083
Diseases of the blood and blood-forming organs	129 (1.0)	108 (1.0)	21 (1.3)	0.233
Diseases of the sense organs (e.g., eyes, ears)	95 (0.8)	90 (0.8)	5 (0.3)	0.027
Mental disorders	66 (0.5)	64 (0.6)	2 (0.1)	0.017
Complications of medical care	42 (0.3)	35 (0.3)	7 (0.4)	0.451
Complications of pregnancy, childbirth, and the puerperium	0 (0)	0 (0)	0 (0)	-
Congenital anomalies	0 (0)	0 (0)	0 (0)	-
Certain conditions originating in the perinatal period	0 (0)	0 (0)	0 (0)	-

**Table 3:** Baseline characteristics in relation to acute kidney injury categories according to KDIGO classification.

	Total	No AKI	AKI
Number of patients	12555	10949	1606
*Demographics*			
Age, year	63 ± 18.5	61 ± 18.8	69 ± 15.3
Gender, men (%)	6755 (53.8)	5932 (54.2)	8273 (51.2)
BMI, kg/m^2^	23.9 ± 5.7	24.0 ± 5.6	23.4 ± 6.0
BMI, kg/m^2^ by categories (%)			
<18.5	1727 (13.8)	1452 (13.3)	275 (17.1)
18.5–23	4339 (34.6)	3775 (34.5)	564 (35.1)
23–27.5	3902 (31.1)	3414 (31.2)	488 (30.4)
>27.5	2587 (20.6)	2308 (21.1)	279 (17.4)
*Ethnicity (*%)			
Chinese	7776 (61.9)	6755 (61.7)	1021 (63.4)
Malay	2360 (18.8)	2033 (18.6)	327 (20.4)
Indian	1485 (11.8)	1334 (12.2)	151 (9.4)
Others	934 (7.4)	827 (7.6)	107 (6.7)
*Specialty (*%)			
Medicine	6353 (50.6)	5524 (50.4)	829 (51.6)
Surgery	1679 (13.4)	1478 (13.5)	201 (12.5)
Geriatrics	1591 (12.7)	1302 (11.9)	289 (18.0)
Cardiology	1479 (11.8)	1301 (11.9)	178 (11.0)
Orthopedic surgery	1147 (9.1)	1084 (9.9)	63 (3.9)
Urology	248 (2.0)	205 (1.9)	43 (2.7)
Otolaryngology	41 (0.3)	39 (0.4)	2 (0.1)
Ophthalmology	17 (0.1)	16 (0.1)	1 (0.1)
*Background diagnosis (*%)			
Hypertension	4895 (39.0)	4045 (36.9)	850 (52.9)
Dyslipidemia	3896 (31.0)	3255 (29.7)	641 (39.9)
Diabetes mellitus	3289 (26.2)	2644 (24.1)	645 (40.2)
Cardiovascular disease	2515 (20)	2004 (18.3)	511 (31.8)
Orthopedic related disease	1402 (11.2)	1146 (10.5)	256 (15.9)
Cerebrovascular disease	1314 (10.5)	1053 (9.6)	261 (16.3)
Neurological disease	1219 (9.7)	1003 (9.2)	216 (13.4)
Respiratory disease	1030 (8.2)	899 (8.2)	131 (8.2)
Hematological disease	955 (7.6)	739 (6.7)	216 (13.4)
Gastrointestinal disease	936 (7.5)	787 (7.2)	149 (9.3)
Chronic kidney disease	857 (6.8)	615 (5.6)	242 (15.1)
Psychiatric illness	760 (6.1)	626 (5.7)	134 (8.3)
Urological disease	595 (4.7)	465 (4.2)	130 (8.1)
Underlying malignancy	584 (4.7)	459 (4.2)	125 (7.8)
Hepatobiliary disease	463 (3.7)	350 (3.2)	113 (7.0)
*Procedures (*%)			
Endoscopy	2355 (18.8)	1941 (17.7)	414 (25.8)
Orthopedic surgery	1222 (9.7)	1072 (9.8)	150 (9.3)
Cardiology related procedures (non-open-heart surgery)	584 (4.7)	538 (4.9)	46 (2.9)
Excision of skin lesions	851 (6.8)	734 (6.7)	117 (7.3)
Open laparotomy	579 (4.6)	468 (4.3)	111 (6.9)
Urology related surgery	510 (4.1)	406 (3.7)	105 (6.5)
Laparoscopic surgery (intra-abdominal)	265 (2.1)	243 (2.2)	22 (1.4)
Neurosurgery	159 (1.3)	131 (1.2)	28 (1.7)
Otolaryngology related surgery	149 (1.2)	112 (1.0)	37 (2.3)
Vascular surgery, open	57 (0.5)	49 (4)	8 (0.5)
Laparoscopic assisted bariatric bypass surgery	12 (0.18)	11 (0.1)	1 (0.1)
*Hospitalisation characteristics*			
ICU admission (%)	1638 (13.0)	1204 (11.0)	434 (27.0)
Mortality within 90 days of admission (%)	997 (7.9)	572 (5.2)	425 (26.5)
Length of stay (d, IQR)	5 (3–9)	4 (3–8)	11 (5–21)
*Creatinine measurements*			
Time from baseline measurement to hospitalisation (d, IQR)	71 (29.3–147)	67 (26–144)	79 (37.3–155.5)
Number of measurements within 365 days before hospitalisation (*n*, IQR)	4 (2–6)	4 (2–6)	5 (3–8)

AKI: acute kidney injury; ICU: intensive care unit; BMI: body mass index; IQR: interquartile range.

**Table 4:** Multivariate logistic regression models using bootstrap method for the risk of acute kidney injury in hospitalised patients.

	Odds ratio	95% CI	*P* value
Age (reference: <55)			
55–75	1.91	1.64–2.24	<0.001
>75	2.24	1.89–2.65	<0.001
Gender (reference: female)			
Male	0.96	0.85–1.07	0.43
Ethnicity (reference: Chinese)			
Indian	0.81	0.67–0.98	0.03
Malay	1.18	1.02–1.36	0.02
Background diagnoses			
Cardiovascular disease	1.53	1.35–1.75	<0.001
Chronic kidney disease	2.01	1.73–2.45	<0.001
Diabetes mellitus	1.60	1.42–1.80	<0.001
Hepatobiliary disease	1.71	1.36–2.15	<0.001
Psychiatric illness	1.40	1.14–1.72	<0.001
Gastrointestinal disease	0.83	0.68–1.00	0.058
Primary diagnoses			
Infectious and parasitic diseases	2.08	1.72–2.49	<0.001
Neoplasm	1.53	1.17–1.98	0.002
Diseases of the genitourinary system	1.93	1.62–2.30	<0.001
Diseases of the musculoskeletal system and connective tissue	0.69	0.54–0.88	0.002
Symptoms, signs, and ill-defined conditions	0.63	0.51–0.76	<0.001
Procedures			
Otolaryngology related surgery	2.91	1.94–4.28	<0.001
Endoscopy	1.30	1.13–1.48	<0.001
Open laparotomy	1.56	1.23–1.97	<0.001
Cardiology related percutaneous procedures	0.64	0.46–0.87	0.007

BMI: body mass index; CI: confidence interval. The *P* value of Hosmer-Lemeshow's goodness-of-fit test is 0.35.

## Supplementary Materials

Supplementary Materials Baseline characteristics of hospital admissions according to the presence or absence of creatinine measurement.
