# Supplementary material for: Low, rather than High, Body Mass Index Is a Risk Factor for Acute Kidney Injury in Multiethnic Asian Patients: A Retrospective Observational Study
Source: Int J Nephrol. 2018 Jan 9;2018:3284612. doi: 10.1155/2018/3284612 (PMC5818948; doi:10.1155/2018/3284612)
Supplement: Supplementary Materials — Baseline characteristics of hospital admissions according to the presence or absence of creatinine measurement. [file 3284612.f1.docx]

Supplementary material

Baseline characteristics of hospital admissions according to presence or absence of creatinine measurement

|  | Number | Admission with Creatinine | Number | Admission without Creatinine |
| --- | --- | --- | --- | --- |
| Age, year | 18661 | 66 (53-78) | 16813 | 53 (37-68) |
| Gender, men (%) | 18661 | 10143 (54.3) | 16813 | 10926 (58.7) |
| **Ethnicity (%)** |  |  | 16813 |  |
| Chinese | 18661 | 11489 (61.5) | 16813 | 11019 (59.2) |
| Malay | 18661 | 4763 (25.5) | 16813 | 3369 (18.1) |
| Indian | 18661 | 2297 (12.3) | 16813 | 2624 (14.1) |
| Others | 18661 | 112 (0.6) | 16813 | 1601 (8.6) |
| **Specialty (%)** |  |  | 16813 |  |
| Medicine | 18661 | 936 (50.1) | 16813 | 7632 (41.0) |
| Surgery | 18661 | 2375 (12.7) | 16813 | 3313 (17.8) |
| Geriatrics | 18661 | 2542 (13.6) | 16813 | 1024 (5.5) |
| Cardiology | 18661 | 2334 (12.0) | 16813 | 1712 (9.2) |
| Orthopedic surgery | 18661 | 1475 (7.9) | 16813 | 2513 (13.5) |
| Urology | 18661 | 380 (2.0) | 16813 | 409 (2.2) |
| Otolaryngology | 18661 | 65 (0.3) | 16813 | 1247 (6.7) |
| Ophthalmology | 18661 | 24 (0.1) | 16813 | 149 (0.8) |
| Others | 18661 | 70 (0.4) | 16813 | 614 (3.3) |
| Length of Stay (d, IQR) | 18661 | 5 (3-9) | 16813 | 2 (1-3) |
| BMI, kg/m^2^ | 16607 | 23 (20-26.6) | 13718 | 23.8 (20.8-27.3) |

BMI: body mass index; specialty under “others” included dental surgery, emergency department admission for more than 24 hours but not admitted to any of the other specialties and hospice care
